# Myelodysplastic Syndromes and Myelodysplastic Syndromes/Myeloproliferative Neoplasms: A Real-World Experience From a Developing Country

**DOI:** 10.1200/GO.23.00281

**Published:** 2024-02-29

**Authors:** Abdalla Awidi, Marah Alzu'bi, Nada Odeh, Jawad Alrawabdeh, Muntaser Al Zyoud, Yazan Hamadneh, Hisham Bawa'neh, Ahmad Magableh, Alaa Alshorman, Feras Al-Fararjeh, Tariq Aladily, Amer M. Zeidan

**Affiliations:** ^1^ Medical School, University of Jordan, Amman, Jordan; ^2^ Jordan University Hospital, Amman, Jordan; ^3^ Cell Therapy Center, University of Jordan, Amman, Jordan; ^4^ Al-Basheer Hospital, Ministry of Health, Amman, Jordan; ^5^ Yale Cancer Center and Smilow Cancer Hospital, Yale University School of Medicine, New Haven, CT

## Abstract

**PURPOSE:**

Myelodysplastic syndromes (MDS) include a heterogeneous group of clonal bone marrow disorders characterized by ineffective hematopoiesis. They manifest as dysplasia in bone marrow hemopoietic elements associated with peripheral cytopenias with variable risk of AML transformation.

**PATIENTS AND METHODS:**

We analyzed retrospectively registry data collected prospectively from patients with primary MDS and patients with MDS/myeloproliferative neoplasm (MPN) in the Jordan University Hospital between January 2007 and September 2021. The registry captured epidemiologic information such as date of diagnosis, age, gender, date of AML transformation, cytogenetics, MDS subtype, risk group according to Revised International Prognostic Scoring System, and survival. The registry also captured baseline ferritin, B12, and lactate dehydrogenase levels.

**RESULTS:**

A total of 112 patients with MDS and MDS/MPN were included in the registry. Median age at diagnosis was 59 years. The male-to-female ratio was about 1.2. In a multivariate cox regression model, baseline serum ferritin significantly affected survival as patients with levels exceeding 1,000 μg/L had a risk of death three times higher compared with those with <1,000 μg/L levels (*P* < .05).

**CONCLUSION:**

To our knowledge, our study is the first comprehensive study examining the epidemiology and prognostic factors in patients with MDS and patients with MDS/MPN in Jordan. Our results show that MDS and MDS/MPN epidemiology in Jordan is different compared with Western countries. Our results also show that baseline serum ferritin levels can be used as a prognostic marker for patients with MDS.

## Teaser

A cohort of patients with MDS and patients with MDS/MPN in Jordan, a developing country, with 10+ year follow-up.

## INTRODUCTION

Myelodysplastic syndromes (MDS) include a group of clonal bone marrow disorders that are dominated by ineffective hematopoiesis and variable degrees of cytopenias. They have variable risk of AML transformation.

**CONTEXT**

**Key Objective**
What are the epidemiologic, clinical, and prognostic factors for myelodysplastic syndromes (MDS) and myelodysplastic syndromes/myeloproliferative neoplasms (MDS/MPN) in Jordan?
**Knowledge Generated**
Our results show that patients with MDS in Jordan are younger than Western patients with MDS. MDS was more common in males with a male-to-female ratio of 1.2. Of the patients, 11.6% had AML transformation. We also show that baseline serum ferritin levels >1,000 μg/L at diagnosis had worse prognosis, even after adjusting for Revised International Prognostic Scoring System and other clinical factors.
**Relevance**
Our data show the difference between Eastern and Western patients with MDS, highlighting the importance of studying different populations with MDS. To our knowledge, our data also pave the way for further studies to improve the risk stratification and treatment outcomes of patients with MDS.

MDS are more common in older males, with a median age at diagnosis of 76 years in the United States and 74 years in Europe.^1,2^ Patients with MDS are at risk of progression to AML, which occurs in 20%-30% of patients.^3^ Because of ineffective hematopoiesis, peripheral blood cytopenias arise, with anemia being the most frequent cytopenia. About 80% of patients have anemia at diagnosis. Patients with MDS experience a highly variable clinical course, ranging from slow to aggressive progression.

Prognostic scoring systems were devised and developed over time to determine MDS prognosis. These systems include the WHO prognostic scoring system, the International Prognostic Scoring System (IPSS), and the Revised International Prognostic Scoring System (IPSS-R). Stratification of patients into risk groups using these systems is based on a multitude of factors, including hemoglobin level, severity of cytopenias, bone marrow blast cell percentage, and cytogenetic abnormalities.^4-6^ Together, the WHO criteria and the IPSS-R provide an assessment of MDS prognosis.^5,6^

These prognostic systems emphasize the importance of cytogenetics in categorizing the risk of patients with MDS, since about 50% of patients exhibit an abnormal karyotype that harbors various chromosomal aberrations. Each of these abnormalities is associated with variable clinical presentations and disease severity.^7,8^

Myelodysplastic syndromes/myeloproliferative neoplasms (MDS/MPN) are disorders characterized by both myelodysplastic and myeloproliferative features, which prevent them from being discreetly classified as MDS or MPN. MDS/MPN, according to the WHO 2016 classification, include chronic myelomonocytic leukemia (CMML) which is the most common, atypical chronic myeloid leukemia, juvenile myelomonocytic leukemia, and myelodysplastic/MPN unclassifiable (MDS/MPN-U).^5,9,10^

Anemia is common in patients with MDS. It may respond to erythropoietin-stimulating agents and activin receptor-ligand traps. However, patients may require transfusions regularly as the disease progresses, as 80%-90% of patients with MDS receive blood transfusions at some point in their clinical course. This puts them at high risk of transfusion-induced iron overload as indicated by high serum ferritin (SF) levels.^11^ Furthermore, iron overload in MDS may start before patients become transfusion-dependent because of ineffective erythropoiesis that suppresses hepcidin production and increases intestinal iron absorption.^12^ Moreover, chronic inflammatory reaction results in a high SF level, which is commonly observed in MDS.^13^ Iron-chelating agents may be of help in ameliorating the iron overload and its manifestations. This has been associated with a reduction in morbidity and mortality.^14-17^

In this study, we describe the epidemiology of MDS and MDS/MPN in Jordan, which is a developing country. We aim to describe the characteristics of patients who had AML transformation and the prognostic role of SF levels at diagnosis. Additionally, we analyzed mortality and leukemia-free survival over a long time period in patients with MDS who were risk-classified using the IPSS-R risk classification.

## PATIENTS AND METHODS

### Study Population and Design

Data in the registry prospectively captured information such as date of diagnosis, age, sex, AML transformation, bone marrow cytogenetics by both fluorescent in situ hybridization (FISH) and G banding, MDS subtype according to WHO classification, and IPSS-R risk group, and survival. The registry also captured blood count data, baseline serum data such as ferritin, B12 levels, and lactate dehydrogenase (LDH) levels. Accordingly, prognostic scores were calculated for every patient and then were categorized into five risk groups: very low, low, intermediate, high, and very high.

The data were collected over the span of 14 years with 112 entries. Data collection started on January 1, 2007, and ended on September 30, 2021. Data from some patients who were diagnosed before January 2007 were captured if the data at the time of diagnosis were complete.

### IRB Approval and Informed Consent

This study was approved by the institutional review board at the Jordan University Hospital (JUH). A written informed consent was obtained from all participants. The consent form was in accordance with the latest version of the Declaration of Helsinki. All methods were carried out in accordance with the approved guidelines of JUH.

### Data Analysis and Statistics

SPSS version 26 (SPSS Inc, Chicago, IL) was used to analyze the data. Descriptive statistics were used to explore and describe the sample. Univariate and multivariate Cox regression were conducted between ferritin and overall survival (OS). For multivariate Cox regression, we adjusted for age at diagnosis, disease subtype, LDH, sex, and IPSS-R. Additionally, Kaplan-Meier survival analysis was conducted to assess survival on the basis of IPSS-R grouping. For comparisons between categorical variables, we used both the chi square test and Fischer's exact test. Finally, Little's Missing Completely at Random test was conducted to assess whether our data were missing completely at random.

### Cytogenetic Analysis

Conventional cytogenetic testing was outsourced, and the karyotypes were reported according to the 2005 International System for Human Cytogenetic Nomenclature. Bone marrow samples were also tested by G banding and FISH for trisomy 8, deletion 7, deletion 5, and other cytogenetic abnormalities.

### Treatment Data

Because of issues in the hospital database, we were unable to retrieve individual detailed treatment data. To compensate for this, we added details about our protocol for treating MDS in Jordan (Appendix 1)

## RESULTS

### General Patient Characteristics

There were 112 patients with MDS and MDS/MPN recorded in the registry. The male-to-female ratio was 1.196. The median age at diagnosis was 59 years for the whole group. The mean age was 56.44 (±1.694) years. The age range was 72 (14-86) years. Males' median age at diagnosis (54.5 years) was significantly higher than females' median age at diagnosis (45.5 years; *P* < .01). The number of transfusion-dependent and transfusion-independent patients was roughly similar. The median and mean follow-up durations were 27.6 months and 43.08 months, respectively. Follow-up duration ranged from 0.5 months to 193.5 months. All characteristics are found in Table 1.

**TABLE 1:** Baseline Characteristics of Patients

Characteristic	Count (%)
Sex	
Female	51 (45.5)
Male	61 (54.5)
Age, years	Median: males (54.5) females (45.5)
Recorded deaths	50 (44.7)
Transfusion requirements	
Dependent^a^	53 (47.3)
Independent	59 (52.7)
Follow-up duration, months	
0-24	51 (46.8)
24-48	20 (18.3)
48+	38 (34.9)

NOTE. Age was not available for three patients.

^a^ Patient had regular transfusions as defined by Germing et al (2 or more units of RBCs needed per 28 days).^18^

### Classification and Cytogenetics

The most common subtype of MDS among our patients was the single lineage dysplasia (MDS-SLD) forming 25.9%, followed by multilineage dysplasia (MDS-MLD; 23.2%). All classification details are shown in Table 2. Cytogenetic groups were determined on the basis of the IPSS-R classification. Most of our patients (76.3%) were classified as having a good cytogenetic group. All cytogenetic groups are shown in Table 3**.**

**TABLE 2:** Subtypes of MDS According to the WHO-2016 Classification

Classification	Count (%)
CMML	12 (10.7)
MDS with isolated 5q	4 (3.6)
MDS EB1	13 (11.6)
MDS EB2	21 (18.8)
MDS MLD	26 (23.2)
MDS RS	4 (3.6)
MDS SLD	29 (25.9)
MDS-U	1 (0.9)
MDS/MPN-U	1 (0.9)
MDS/MPN-RS-T	1 (0.9)

Abbreviations: CMML, chronic myelomonocytic leukemia; MDS, myelodysplastic syndrome; MDS EB1, MDS excess blasts type 1; MDS EB2, MDS excess blasts type 2; MDS MLD, MDS multilineage dysplasia; MDS RS, MDS ring sideroblasts; MDS SLD, MDS single lineage dysplasia; MDS-U, unclassified MDS; MDS-MPN-U, myelodysplastic-myeloproliferative neoplasm unclassified; MDS-MPN-RS-T, MDS/MPN-ring sideroblasts with thrombocytosis.

**TABLE 3:** Cytogenetic Groups of Patients With MDS as Defined by IPSS-R

Cytogenetics	Count (%)
Good	71 (76.3)
Intermediate	4 (4.3)
Poor	7 (7.5)
Very poor	8 (8.6)

NOTE. Twenty-two patients did not have cytogenetics available.

Abbreviations: IPSS-R, Revised International Prognostic Scoring System; MDS, myelodysplastic syndromes.

### IPSS-R and Survival

In our study, 38.7% of our patients were labeled as low-risk group, making it the most common among our patients. Following the low-risk group, high- and very high-risk groups made up 28% of patients, with very low- and intermediate-risk groups contributing the least to the sample. Details are shown in Table 4.

**TABLE 4:** IPSS-R Risk Groups of Patients

IPSS-R	Count (%)
Very high	10 (10.8)
High	16 (17.2)
Intermediate	9 (9.7)
Low	36 (38.7)
Very low	14 (15.1)

NOTE. IPSS-R risk could not be calculated for 27 patients due to missing variables.

Abbreviations: IPSS-R, Revised International Prognostic Scoring System.

**FIG 1:**
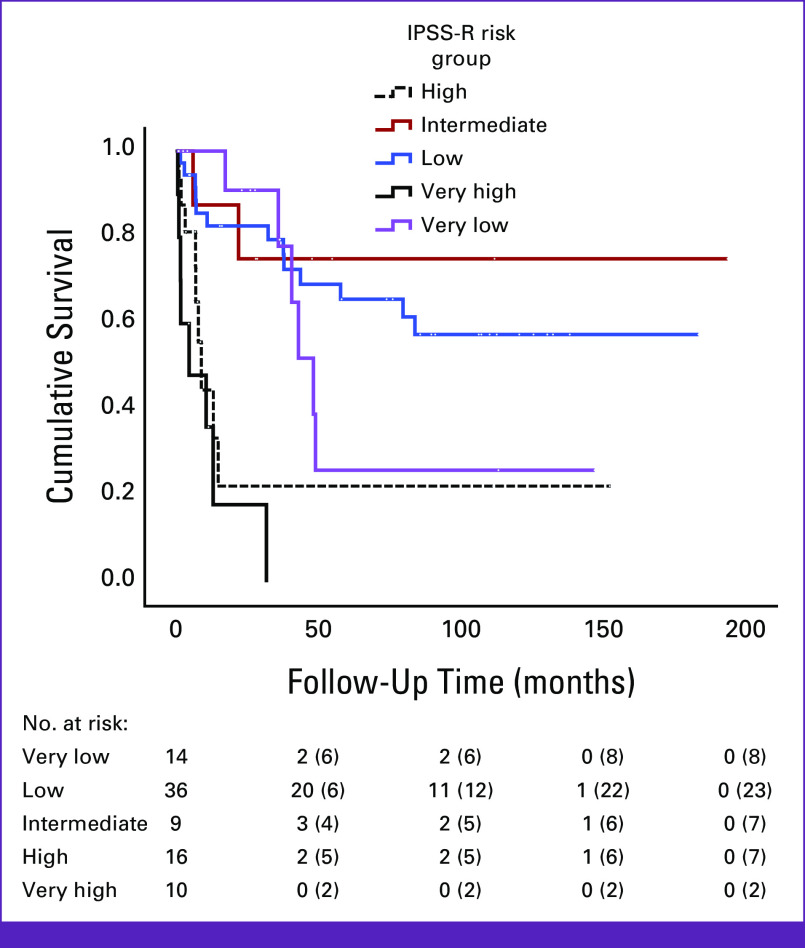
Kaplan-Meier curve of patients with MDS on the basis of IPSS-R risk groups. IPSS-R, Revised International Prognostic Scoring System; MDS, myelodysplastic syndrome.

Kaplan-Meier survival curves of patients with MDS according to IPSS-R risk group are shown in Figure 1. Patients classified as high or very high risk had the shortest OS, followed by patients classified as very low risk.

### The Prognostic Role of Ferritin in Patients With MDS

**FIG 2:**
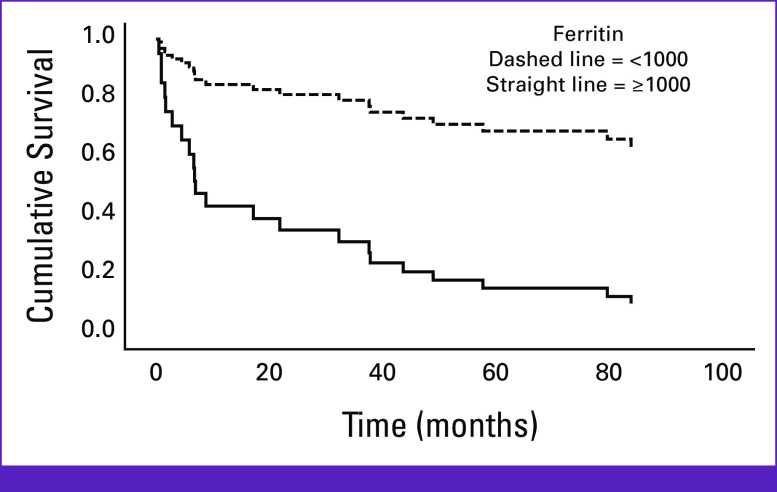
Survival according to ferritin levels (*P* < .01).

Univariate cox regression (excluding patients with MDS/MPN along with those who transformed to AML) showed that baseline SF levels exceeding 1,000 μg/L were associated with unfavorable prognosis (*P* < .01; Fig 2). To ensure it is not associated with transfusion burden, a Cox regression model was done with SF and transfusion status. SF was significantly associated with mortality after adjusting for transfusion status; however, transfusion burden was not significantly associated with mortality (*P* > .05). To ensure the validity of our conclusions, we adjusted for confounders and imputed missing data. The confounders we adjusted for were age at diagnosis, disease subtype, LDH, sex, and IPSS-R in a multivariate analysis. Even so, ferritin still showed significance as patients with baseline SF levels exceeding 1,000 μg/L were more than three times more likely to die in comparison with patients whose SF levels were below 1,000 μg/L. Hazard ratios and *P* values for all univariate and multivariate analyses are shown in Table A1.

### AML Transformation

Of 112 patients, 13 cases were recorded to have transformed to AML. Fisher's exact test showed that those initially diagnosed with MDS-excess blasts type 2 (EB2) accounted for most of the patients with AML (*P* < .01). It also showed that more males transformed to AML in comparison with females (*P* < .05). Patients with AML were more likely to have SF levels exceeding 1,000 μg/L at diagnosis (*P* = .033, risk ratio, 4.138 [95% CI, 1.196 to 15.591]).

### Patients With MDS/MPN

A total of 15 patients were diagnosed with MDS/MPN, 12 of whom had CMML. We also had two cases of MDS/MPN-U and one case of MDS/MPN-ring sideroblasts (RS) with thrombocytosis. For the 12 patients with CMML patients, the average follow-up duration was 40.55 (1.71-132.24) months. Four patients were female. The mean age was 55.67 years. Eight patients died with a median OS time of 38.86 months. Two patients had AML transformation.

## DISCUSSION

The epidemiology of MDS has been shown to differ between countries. Jordan as an example has been shown to have different epidemiologic patterns compared with other countries. This highlights the importance of studying MDS epidemiologic patterns. This case is especially evident in the Middle East and North Africa (MENA) region as it is understudied in these regions. This study is an update of our previous work on MDS.^18-20^

There are four main findings of this study. First, the mean age for our study sample was 56.45 years, similar to our previous study on MDS.^20^ This age is strikingly younger than the age reported in many previous studies in Western population.^21-23^ Many recent studies from the MENA region reported mean age values close to the one reported in our study. The mean age values of MDS populations in Saudi Arabia and Egypt were 50 and 56 years, respectively,^24,25^ whereas the mean age values for the United States, Germany, and Greece were 77, 71, and 75 years, respectively.^26-28^ MDS has also been shown to begin at a younger age in Asian countries, including Japan, China, Korea, India, Thailand, and Turkey.^29^ The reason for this younger age is still unknown, although it might be due to the fact that people age 65 years or older form only around 5% of the total population in many of the MENA countries.^30^ In addition, Jordan has a slightly lower life expectancy in comparison with Western countries, which might have contributed to the younger median age for the MDS population in Jordan. According to World Bank's reports for 2020, males had a life expectancy of 73 years and females had a life expectancy of 78 years. These ages were slightly higher than those for the MENA region, with 71 years and 75 years for males and females, respectively. Life expectancy values in Western countries were higher. The life expectancies for males and females in the United States were 75 years and 80 years, respectively. Also, the life expectancies for males and females in the European Union countries were 80.5 years and 83.2 years, respectively. However, life expectancy difference alone is not enough to explain the huge magnitude of change between Eastern and Western countries. In addition to the possible justifications listed above, we concur that possible hereditary, genetic, immunologic, infectious, and other environmental factors may have contributed to the age difference at diagnosis between world regions.^29,31^

Second, male patients with MDS are more likely to progress to AML when compared with females. This is consistent with previous literature. MDS showed predominance in males in both Western countries and the MENA region as studies from both parties reported male-to-female ratios ranging from 1.6 to 2.^21-25^ A number of studies in the past 3 years revealed that males with MDS have worse OS and are more prone to transformation to AML in comparison with females.^26,32,33^ One possible justification for the increased transformation to AML among males is higher smoking rates.^34-36^ Third, we found that SF levels influence OS of non-AML MDS patients. Patients with MDS with baseline SF levels >1,000 μg/L had poorer prognosis when compared with those with levels <1,000 μg/L. This result is consistent with the work by Pileggi et al^37^ that reported the effect of elevated SF levels on the OS of patients with MDS. It is worth mentioning that AML cases were excluded as they had very poor prognosis regardless of SF levels. Fourth, both groups of patients with MDS, those who were initially diagnosed with MDS-EB2 and those with ferritin levels exceeding 1,000 μg/L, were more likely to progress to AML. Iron overload has been discussed to be involved in mutagenesis and leukemic transformation, but data are still ambiguous and more prospective trials are required.^38^ Accordingly, it is possible that iron overload–induced oxidative stress and genetic instability are the fundamental contributors to this finding. It is essential to point out that elevated SF levels may indicate an acute or chronic inflammatory reaction caused by the disease itself when these elevated levels cannot be explained by high transfusion burden. Such inflammatory reactions have been linked to worse prognosis and higher risk of developing AML in patients with MDS. We studied the effect of SF levels at diagnosis to make sure it was not an effect of high transfusion burden. Unfortunately, the registry used in the present work has not captured data on other serum inflammatory markers or iron overload markers besides ferritin, which makes it a bit more difficult to ensure absolute causation. Our data show that of a total of 112 patients with MDS, only 11.6% progressed to AML versus 30% as reported in the literature. It may be possible that the younger age in our population accounts for this low rate of AML transformation.^39-42^

One surprising finding in our study is that patients with very low risk disease by IPSS-R had a relatively short survival. Recently, research into the incorporation of somatic mutations into prognostic evaluations has shown the capacity to further stratify patients regarding leukemic transformation and OS. As many low-risk patients with MDS were restratified into high risk after accounting for these mutations. Accordingly, the authors suggest a modified risk score for patients with MDS which includes these mutations. We did not specifically look at these mutations in detail as next-generation sequencing for patients with MDS in Jordan was only very recently available, but it may be possible that some of our patients harbor these high-risk mutations. This would explain our finding. Nonetheless, more studies are needed to clarify this finding.^43,44^

A recent systematic literature review and meta-analysis evaluated the association between SF levels and clinical outcomes in patients with myelodysplastic syndromes (MDS). The review identified 21 observational studies measuring the relationship between SF levels and survival outcomes such as OS, event-free survival, and relapse-free survival in over 2000 patients with MDS. Although thresholds and definitions of elevated SF varied between studies, the meta-analysis found that higher SF levels, particularly levels over 1,000 μg/L, were generally associated with worse survival outcomes. These findings are consistent with our results showing that patients with MDS with SF levels >1,000 μg/L had poorer prognosis compared with those with levels <1,000 μg/L.

Another study analyzed a large cohort of 687 higher-risk patients with MDS and oligoblastic AML treated with 5-azacytidine. They found that elevated SF levels (>520 ng/mL) independently predicted poorer response to treatment and inferior OS and leukemia-free survival. Most importantly, they demonstrated that the prognostic value of SF was independent of RBC transfusion need and other markers of inflammation. Our results are consistent with these findings, as we also observed that patients with MDS with baseline SF levels >1,000 μg/L had significantly poorer prognosis compared with those with lower levels. The cutoff value of 1,000 μg/L used in our study corresponds well with the 520 ng/mL cutoff identified by Papageorgiou et al in predicting worse outcomes. Taken together, these findings indicate that SF is an important prognostic factor in MDS, regardless of geographical differences in patient populations. Its incorporation into risk stratification could help identify high-risk patients most likely to benefit from alternative therapeutic approaches.^48,49^

One limitation of this study and the registry itself is that the cause of death (COD) is not captured by the registry. As such, we were unable to know if the COD was directly related to MDS or other comorbidities. We did not inspect the immediate COD for all patients with MDS. Other limitations include the relatively small sample size of our study population; the absence of information on comorbidities, treatment, and treatment outcomes in the used registry; the fairly representative yet incomplete mutation profile for the present study's patients; and the unavailability of some pertinent laboratory blood tests such as mean corpuscular volume, serum c-reactive protein, and transferrin saturation.

Future studies are needed to confirm our findings, including the younger age of onset, the lower-than-expected OS of low-risk patients, and the prognostic role of SF at diagnosis. Early incorporation of iron chelation therapy might be beneficial.^15,45-47^

In conclusion, our work confirms a decent number of the previously reported epidemiologic findings concerned with MDS populations in the MENA region and significantly adds to the body of MDS literature in Jordan. The results of this study state that MDS occurs at a younger age in the Jordan and is more prevalent in males. They also affirm the huge impact of high SF level on the survival of patients with MDS and highlight the shorter survival of low-risk MDS emphasizing on the necessity to incorporate high-risk mutations in the model of survival in addition to the IPSS-R model. This will be studied in detail in further projects.

## APPENDIX 1. SUPPLEMENTARY DATA

### Treatment Protocol for MDS

#### A: Low and Intermediate Risk

1. For anemia in low and intermediate risk, supportive care with careful observation is the initial step. If anemia is symptomatic or hemoglobin is 8 g/dl or less with low serum erythropoietin, then erythropoietin-stimulating agents are used (epoetin alfa or darbepoetin alfa upon availability). Blood transfusion when needed, and if needed iron chelation, may be used. Luspatercept is not registered and not available in the country.
2. Hypomethylating agent or lenalidomide may be used in patients who fail erythropoietin-stimulating agents. If the bone marrow is hypoplastic, antithymocyte globulin with cyclosporine A is used along with granulocyte colony-stimulating factor support.

#### B: High Risk

Hypomethylating agents, induction chemotherapy, erythropoiesis-stimulating agents, lenalidomide, and luspatercept in addition to supportive care are all therapeutic options that can be used for management of patients with MDS according to the risk stratification category and other disease- and patient-related factors. If an actionable mutation is found, the patient is advised to use the drug for the mutation if it is available.

**TABLE A1:** Detailed Results From Univariable and Multivariate Cox Regression

Univariate Analysis			Multivariate Analysis		
Variable	*P*	HR	Variable	*P*	HR
Ferritin >1,000	.001	5	Ferritin >1,000	.043	3.144
Age at diagnosis	.045	1.023	Age at diagnosis	.229	—
Sex	.011	2.71	Sex	.155	—
LDH	.39	—	LDH	.316	—
IPSS-R Very low^a^	.003		IPSS-R Very low^a^	.927	
Low	.668	—	Low	.58	—
Intermediate	.623	—	Intermediate	.535	—
High	.322	—	High	.753	—
Very high	.004	8.778	Very high	.831	—
Classification MDS-SLD^a^	<.00001		Classification MDS-SLD^a^	.074	
MDS-MLD	.363	—	MDS-MLD	.844	—
MDS-EB-1	.006	5.5	MDS-EB-1	.026	—
MDS-EB-2	<.0001	20.5	MDS-EB-2	.056	—
MDS-RS	.236	—	MDS-RS	.342	—

NOTE. *P* values are shown for each variable. HR was reported for significant covariates.

Abbreviations: HR, hazard ratio; IPSS-R, Revised International Prognostic Scoring System; LDH, lactate dehydrogenase; MDS, myelodysplastic syndrome; MDS-EB-1, MDS excess blasts type 1; MDS-EB-2, MDS excess blasts type 2; MDS-MLD, MDS multilineage dysplasia; MDS-RS, MDS ring sideroblasts; MDS-SLD, MDS single lineage dysplasia.

^a^ The reference category for the specific categorical variable.

## References

[1] Ma X, Does M, Raza A, et al: Myelodysplastic syndromes: Incidence and survival in the United States. Cancer 109:1536-1542, 2007 17345612 10.1002/cncr.22570

[2] Stauder R, Yu G, Koinig KA, et al: Health-related quality of life in lower-risk MDS patients compared with age- and sex-matched reference populations: A European LeukemiaNet study. Leukemia 32:1380-1392, 2018 29572506 10.1038/s41375-018-0089-x PMC5990524

[3] Steensma DP, Bennett JM: The myelodysplastic syndromes: Diagnosis and treatment. Mayo Clinic Proc 81:104-130, 2006 10.4065/81.1.104 16438486

[4] Greenberg P, Cox C, LeBeau MM, et al: International scoring system for evaluating prognosis in myelodysplastic syndromes. Blood 89:2079-2088, 1997 9058730

[5] Vardiman JW, Thiele J, Arber DA, et al: The 2008 revision of the World Health Organization (WHO) classification of myeloid neoplasms and acute leukemia: Rationale and important changes. Blood 114:937-951, 2009 19357394 10.1182/blood-2009-03-209262

[6] Greenberg PL, Tuechler H, Schanz J, et al: Revised international prognostic scoring system for myelodysplastic syndromes. Blood 120:2454-2465, 2012 22740453 10.1182/blood-2012-03-420489 PMC4425443

[7] Raza A, Galili N: The genetic basis of phenotypic heterogeneity in myelodysplastic syndromes. Nat Rev Cancer 12:849-859, 2012 23175121 10.1038/nrc3321

[8] Bejar R, Stevenson K, Abdel-Wahab O, et al: Clinical effect of point mutations in myelodysplastic syndromes. N Engl J Med 364:2496-2506, 2011 21714648 10.1056/NEJMoa1013343 PMC3159042

[9] Wang SA, Hasserjian RP, Fox PS, et al: Atypical chronic myeloid leukemia is clinically distinct from unclassifiable myelodysplastic/myeloproliferative neoplasms. Blood 123:2645-2651, 2014 24627528 10.1182/blood-2014-02-553800 PMC4067498

[10] Elliott MA, Hanson CA, Dewald GW, et al: WHO-defined chronic neutrophilic leukemia: A long-term analysis of 12 cases and a critical review of the literature. Leukemia 19:313-317, 2004 10.1038/sj.leu.2403562 15549147

[11] Hellström-Lindberg E: Management of anemia associated with myelodysplastic syndrome. Semin Hematol 42:S10-S13, 2005 (2 suppl 1) 15846579 10.1053/j.seminhematol.2005.01.002

[12] Santini V, Girelli D, Sanna A, et al: Hepcidin levels and their determinants in different types of myelodysplastic syndromes. PLoS One 6:e23109, 2011 21886780 10.1371/journal.pone.0023109 PMC3158762

[13] Barreyro L, Chlon TM, Starczynowski DT: Chronic immune response dysregulation in MDS pathogenesis. Blood 132:1553-1560, 2018 30104218 10.1182/blood-2018-03-784116 PMC6182269

[14] Poggiali E, Cassinerio E, Zanaboni L, et al: An update on iron chelation therapy. Blood Transfus 10:411-422, 2012 22790257 10.2450/2012.0008-12 PMC3496216

[15] Zeidan AM, Hendrick F, Friedmann E, et al: Deferasirox therapy is associated with reduced mortality risk in a medicare population with myelodysplastic syndromes. J Comp Eff Res 4:327-340, 2015 26274794 10.2217/cer.15.20 PMC5549705

[16] Musto P, Maurillo L, Simeon V, et al: Iron-chelating therapy with deferasirox in transfusion-dependent, higher risk myelodysplastic syndromes: A retrospective, multicentre study. Br J Haematol 177:741-750, 2017 28419408 10.1111/bjh.14621

[17] Neukirchen J, Fox F, Kündgen A, et al: Improved survival in MDS patients receiving iron chelation therapy—A matched pair analysis of 188 patients from the Düsseldorf MDS registry. Leuk Res 36:1067-1070, 2012 22564985 10.1016/j.leukres.2012.04.006

[18] Germing U, Oliva EN, Hiwase D, et al: Treatment of anemia in transfusion-dependent and non-transfusion-dependent lower-risk MDS: Current and emerging strategies. HemaSphere 3:e314, 2019 31976486 10.1097/HS9.0000000000000314 PMC6924547

[19] Jiang Y, Eveillard JR, Couturier MA, et al: Asian population is more prone to develop high-risk myelodysplastic syndrome, concordantly with their propensity to exhibit high-risk cytogenetic aberrations. Cancers 13:481, 2021 33513838 10.3390/cancers13030481 PMC7865620

[20] Awidi A, Magableh A, Taimeh Z, et al: Primary myelodysplastic syndrome in Jordan: A single-centre experience. Med principles Pract 18:351-355, 2009 10.1159/000226286 19648755

[21] Ma X: Epidemiology of myelodysplastic syndromes. Am J Med 125:S2-S5, 2012 (7 suppl) 10.1016/j.amjmed.2012.04.014 PMC3394456 22735748

[22] Avgerinou C, Alamanos Y, Zikos P, et al: The incidence of myelodysplastic syndromes in Western Greece is increasing. Ann Hematol 92:877-887, 2013 23572136 10.1007/s00277-013-1712-6 PMC3674340

[23] Chihara D, Ito H, Katanoda K, et al: Incidence of myelodysplastic syndrome in Japan. J Epidemiol 24:469-473, 2014 25088696 10.2188/jea.JE20140042 PMC4213221

[24] Elnahass Y, Youssif L: Cytogenetic features in primary myelodysplastic syndrome Egyptian patients. J Adv Res 10:77-83, 2018 30046476 10.1016/j.jare.2018.02.002 PMC6057444

[25] AlMozain N, Mashi A, Alneami Q, et al: Spectrum of myelodysplastic syndrome in patients evaluated for cytopenia(s). A Report from a Reference Centre in Saudi Arabia. Hematol Oncol Stem Cell Ther 15:39-44, 2022 33227261 10.1016/j.hemonc.2020.11.001

[26] Zeidan AM, Shallis RM, Wang R, et al: Epidemiology of myelodysplastic syndromes: Why characterizing the beast is a prerequisite to taming it. Blood Rev 34:1-15, 2019 30314642 10.1016/j.blre.2018.09.001

[27] Matsuda A, Germing U, Jinnai I, et al: Difference in clinical features between Japanese and German patients with refractory anemia in myelodysplastic syndromes. Blood 106:2633-2640, 2005 15972453 10.1182/blood-2005-01-0040

[28] Avgerinou C, Giannezi I, Theodoropoulou S, et al: Occupational, dietary, and other risk factors for myelodysplastic syndromes in Western Greece. Hematology 22:419-429, 2017 28102107 10.1080/10245332.2016.1277006

[29] Paydas S: Young age MDS: Differences between western and eastern countries. Leuk Res 30:362, 2006 10.1016/j.leukres.2005.07.005 16125229

[30] Abyad A: Ageing in the Middle-East and North Africa: Demographic and health trends. Int J Ageing Developing Countries 6:112-128, 2021

[31] Komrokji R: Myelodysplastic syndromes: A view from where the sun rises and where the sun sets. Leuk Res 30:1067-1068, 2006 16723153 10.1016/j.leukres.2006.04.004

[32] Wang F, Ni J, Wu L, et al: Gender disparity in the survival of patients with primary myelodysplastic syndrome. J Cancer 10:1325-1332, 2019 30854142 10.7150/jca.28220 PMC6400681

[33] Karantanos T, Gondek LP, Varadhan R, et al: Gender-related differences in the outcomes and genomic landscape of patients with myelodysplastic syndrome/myeloproliferative neoplasm overlap syndromes. Br J Haematol 193:1142-1150, 2021 34028801 10.1111/bjh.17534 PMC8217263

[34] Alkouri O, Khader Y, Al-Bashaireh AM: Prevalence of cigarettes and waterpipe smoking among Jordanians, refugees, and migrants in Jordan and its associated factors: A secondary data analysis. Int J Environ Res Public Health 20:82, 2022 36612400 10.3390/ijerph20010082 PMC9819960

[35] Mishra A, Rollison DE, Brandon TH, et al: Impact of tobacco usage on disease outcome in myelodysplastic syndromes. Leuk Res 39:673-678, 2015 25934048 10.1016/j.leukres.2015.03.020 PMC5992898

[36] Zavras PD, Pophali P, Shastri A, et al: Increased mortality among smokers with myelodysplastic syndrome (MDS). J Clin Oncol 39, 2021 (suppl 15; abstr e19036)

[37] Pileggi C, Di Sanzo M, Mascaro V, et al: Role of serum ferritin level on overall survival in patients with myelodysplastic syndromes: Results of a meta-analysis of observational studies. PLoS One 12:e0179016, 2017 10.1371/journal.pone.0179016 PMC5473533 28622367

[38] Mishra A, Corrales-Yepez M, Ali NA, et al: Validation of the revised International Prognostic Scoring System in treated patients with myelodysplastic syndromes. Am J Hematol 88:566-570, 2013 23605934 10.1002/ajh.23454 PMC4685468

[39] Chai X, Li D, Cao X, et al: ROS-mediated iron overload injures the hematopoiesis of bone marrow by damaging hematopoietic stem/progenitor cells in mice. Sci Rep 5:10181, 2015 25970748 10.1038/srep10181 PMC4429544

[40] Pilo F, Angelucci E: A storm in the niche: Iron, oxidative stress and haemopoiesis. Blood Rev 32:29-35, 2018 28847531 10.1016/j.blre.2017.08.005

[41] Westhofen G, Ganster C, Beier F, et al: Comprehensive genomic analysis provides further evidence that iron overload can induce genetic instability in myelodysplastic syndromes. Blood 126:2842, 201526491069

[42] Menssen AJ, Walter MJ: Genetics of progression from MDS to secondary leukemia. Blood 136:50-60, 2020 32430504 10.1182/blood.2019000942 PMC7332895

[43] Madry K, Yu G, Lis K, et al: Excess mortality in low-risk MDS can be explained by MDS and AML related causes of death. Blood 132:4385, 2018 (suppl 1)

[44] F Al-Tameemi W, Qasim Ibrahim S: Overview of myelodysplastic syndrome in Baghdad, Iraq- clinical picture and outcome. Hematol Transfus Int J 9:75-81, 2021

[45] Mitchell M, Gore SD, Zeidan AM: Iron chelation therapy in myelodysplastic syndromes: Where do we stand? Expert Rev Hematol 6:397-410, 2013 23991926 10.1586/17474086.2013.814456 PMC4124619

[46] Zeidan AM, Griffiths EA: To chelate or not to chelate in MDS: That is the question. Blood Rev 32:368-377, 2018 29602612 10.1016/j.blre.2018.03.002

[47] Zeidan AM, Giri S, DeVeaux M, et al: Systematic review and meta-analysis of the effect of iron chelation therapy on overall survival and disease progression in patients with lower-risk myelodysplastic syndromes. Ann Hematol 98:339-350, 2019 30413901 10.1007/s00277-018-3539-7

[48] Oliva EN, Huey K, Deshpande S, et al: A systematic literature review of the relationship between serum ferritin and outcomes in myelodysplastic syndromes. J Clin Med 11:895, 2022 35160344 10.3390/jcm11030895 PMC8836890

[49] Papageorgiou SG, Kotsianidis I, Bouchla A, et al: Serum ferritin and ECOG performance status predict the response and improve the prognostic value of IPSS or IPSS-R in patients with high-risk myelodysplastic syndromes and oligoblastic acute myeloid leukemia treated with 5-azacytidine: A retrospective analysis of the Hellenic National registry of myelodysplastic and hypoplastic syndromes. Ther Adv Hematol 11:204062072096612, 2020 10.1177/2040620720966121 PMC7727043 33343854

